# Ontogenetic Survey of Histone Modifications in an Annelid

**DOI:** 10.1155/2012/392903

**Published:** 2012-02-19

**Authors:** Glenys Gibson, Corban Hart, Robyn Pierce, Vett Lloyd

**Affiliations:** ^1^Department of Biology, Acadia University, 33 Westwood Avenue, Wolfville, NS, Canada B4P 2R6; ^2^Department of Biology, Mount Allison University, 63B York Street, Sackville, NB, Canada E4L 1G7

## Abstract

Histone modifications are widely recognized for their fundamental importance in regulating gene expression in embryonic development in a wide range of eukaryotes, but they have received relatively little attention in the development of marine invertebrates. We surveyed histone modifications throughout the development of a marine annelid, *Polydora cornuta*, to determine if modifications could be detected immunohistochemically and if there were characteristic changes in modifications throughout ontogeny (surveyed at representative stages from oocyte to adult). We found a common time of onset for three histone modifications in early cleavage (H3K14ac, H3K9me, and H3K4me2), some differences in the distribution of modifications among germ layers, differences in epifluorescence intensity in specific cell lineages suggesting that hyperacetylation (H3K14ac) and hypermethylation (H3K9me) occur during differentiation, and an overall decrease in the distribution of modifications from larvae to adults. Although preliminary, these results suggest that histone modifications are involved in activating early development and differentiation in a marine invertebrate.

## 1. Introduction

One of the central questions in biology is how differences in gene expression during development lead to the generation of form. Epigenetic mechanisms such as histone modifications activate or silence gene expression and thereby provide rapid, reversible mechanisms that regulate gene expression in embryonic development. The importance of histone modifications in development has been extensively studied in model systems. As this approach is gradually extended to nonmodel species, histone modifications are being discovered as mechanisms that are highly conserved in a wide variety of eukaryotes and critically important in regulating fundamental developmental processes, including meiosis [1], cell differentiation [2], organ development in plants [3], sexual and asexual reproduction in fungi [4], genomic imprinting in plants and insects [5], and X-inactivation in mammals [6]. 

Despite the clearly established importance of histone modifications in the development of many eukaryotes, they have received almost no attention in the development of benthic marine invertebrates. Benthic marine invertebrates represent an exciting group for epigenetic research as they not only are morphologically diverse as adults, but their larvae are morphologically and behaviorally distinct from adults and form the basis for an impressive diversity of life-history patterns. Our objectives are to determine if histone modifications can be detected in a marine worm using immunohistochemistry, if modifications differ among differentiating tissues, and if changes in modifications correlate with ontogenetic transitions. We chose the worm *Polydora cornuta* Bosc, 1802 (Annelida, Spionidae) for this study. *P. cornuta* is a small, opportunistic detritivore that is common in intertidal mudflats and has a wide distribution in temperate and subtropical coastal areas [7, 8]. Fertilization in *P. cornuta* is internal, and females deposit zygotes in a string of egg capsules that they brood in their mud tubes. Larval development for this species has been described by several authors and is strongly influenced by the presence of nurse eggs in the egg capsules [8–12]. Some broods contain only a few nurse eggs and young hatch as small, swimming larvae that feed on phytoplankton (a trophic mode termed planktotrophy). In other broods, most eggs are nondeveloping nurse eggs which provide extraembryonic nutrition for encapsulated larvae (termed adelphophagy), and as a result, young hatch as large, advanced larvae which settle soon after hatching. Although two developmental morphs are observed for *P. cornuta*, the present study focuses on epigenetic similarities between morphs. Our goal is to establish a foundational understanding of changes in the epigenome throughout development, as the first step in a larger project that investigates the potential for histone modifications to influence plasticity in larval development in this species. 

We surveyed histone modifications throughout ontogeny using immunohistochemistry. The survey included oocytes, embryos, early larvae, and adults. This allowed us to correlate histone modifications with specific developmental events (e.g., completion of meiosis, tissue formation) and life history stages (i.e., embryos, larvae, and adults). We focused on histone modifications as histones are among the most highly conserved proteins in eukaryotes [13]. Their modifications are equally conserved and are an important aspect of epigenetic gene regulation in many different organisms [5, 14–17]. We used antibodies for core histones as well as for four commonly studied histone modifications including antihistone H3 acetyl Lys14 (referred to in this paper as H3K14ac), antihistone H3 dimethyl Lys4 (or H3K4me2), antihistone H3 monomethyl Lys9 (or H3K9me), and antihistone H4 dimethyl Lys20 (or H4K20me2). Generally, H3K14ac is associated with transcription, as acetylation loosens the nucleosomes and allows transcription factors to bind to promoter regions; H3K9me and H3K4me2 are associated with both transcription and gene silencing; H4K20me2 is associated with gene silencing [2, 18–20]. Because we do not know the transcriptional outcome of a change in histone modifications in *Polydora cornuta*, and also because of the overall complexity of the epigenome, we follow the advice of Turner [18, 21] and interpret an ontogenetic change in histone modifications as a change within the histone code, rather than a specific indicator of gene expression. We show that histone modifications were detected throughout ontogeny in *Polydora cornuta*. Similarities in the distribution of three histone modifications suggest that certain phases of development (i.e., early cleavage and possibly metamorphosis) represent transition points during which widespread changes in histone modifications occur. 

## 2. Materials and Methods

### 2.1. Collection and Culture

Adult *Polydora cornuta* were collected from intertidal mudflats at West Marsh, Halifax Co., Nova Scotia (N44.6456, W-63.3744) in early summer (May to July) of 2010 and 2011. Adults were cultured in 250 mL Pyrex crystallizing dishes which contained enough sand to cover the bottom. Each dish contained approximately 10–16 worms, including some males to ensure sperm availability. Cultures were immersed in seawater at approximately 14-15°C, provided with continuous aeration, and maintained on a 15 : 9 LD photoperiod. 

After spawning, broods were removed from the females' tubes and cultured. As *P. cornuta* is poecilogonous [11], broods were identified under a compound microscope by determining the trophic morph of young (planktotrophy or adelphophagy) and counting the number of nurse eggs per egg capsule. We use the term *P-brood* to refer to broods in which there are few or no nurse eggs (<5% of the total number of eggs per brood), most eggs develop (approximately 80 embryos/capsule) and young hatch as small (3 to 5 segments), planktotrophic larvae. The term *A-brood* is used for broods in which most eggs (>90%) are nurse eggs, few young develop (approximately 5/capsule), and most young are adelphophagic while in the egg capsule (data from MacKay and Gibson) [11]. Individual egg capsules were placed in 3.5 mL Falcon well plates containing filtered seawater with antibiotics (1000 mL seawater : 1 mL penicillin-streptomycin; Sigma P4333). Well plates and culture water were changed daily until broods reached desired ontogenetic stages. Stages examined were oocyte, cleavage (2- to 32-cell stages), blastula, gastrula, trochophore, metatrochophore, early larva (3-4 chaetigers), and for adelphophagic morphs only, advanced larvae (5–12 chaetigers). 

Forty-eight A-broods and sixteen P-broods were examined. Unequal sample sizes reflect the fact that P-broods were relatively uncommon in the West Marsh population. Approximately eight egg capsules were fixed per brood per ontogenetic stage. We processed two to three egg capsules per assay and examined all embryos per capsule for consistency in epifluorescence (i.e., presence and relative intensity of epifluorescence in specific cells or tissues). A complete examination was done for young from both A- and P-broods at all ontogenetic stages, and observations of histone modifications common to both morphs are presented.

### 2.2. Fixation and Immunohistochemistry

Embryos and larvae of both morphs were fixed and labeled with commercially available primary antibodies for histones and histone modifications. Egg capsules were fixed in 4% paraformaldehyde in phosphate buffered saline (PBS) for 45 minutes on ice, rinsed in PBS, dehydrated to absolute methanol, and stored at −20°C for up to two months. 

Immunohistochemistry was performed using a procedure modified from Sagawa et al. [22] and kindly provided by Shiga. Specimens were rehydrated to phosphate buffered saline containing 1% (v/v) Tween 20 (PT), and under a dissecting microscope, a small hole was then torn in each egg capsule to allow further reagents to enter. Specimens were blocked in PT containing 2% (w/v) bovine serum albumin (2% BSA/PT) for 2 hours at 4°C and then incubated in one of the following primary antibodies (1/500) in 2% BSA/PT overnight at 4°C. Five primary antibodies were used: antihistone, histone 1 and core histones (monoclonal mouse, Millipore MAB052), antihistone H3 acetyl Lys14 (monoclonal rabbit, Abcam ab52946), antihistone H3 dimethyl Lys4 (polyclonal rabbit, Abcam ab32356), antihistone H3 monomethyl Lys9 (polyclonal rabbit, Abcam ab9045), and antihistone H4 dimethyl Lys20 (polyclonal rabbit, Abcam ab9052-25). Histone proteins and modifications are highly conserved [13], and the primary antibodies used here have also been used to detect the same histone modifications in a wide variety of eukaryotes, ranging from yeast to plants [23–25]. Specimens were then washed ten times over 1 hour with PT. Secondary antibodies were FITC-goat anti-rabbit IgG (1/50, Invitrogen 65-6111) for specimens incubated with H3K14ac, H3K4me2, H3K9me, and H4K20me2; TRITC-goat anti-mouse IgG (1/50; Invitrogen T-2762) for specimens incubated with antihistone. All specimens were also colabeled with DAPI (1/500; Sigma 32670) in 2% BSA/PT for 2 hours at 4°C. Specimens were washed 12 times over 1 hour with PT and mounted onto glass slides using Vectashield (Vector Laboratories H-1000). Coverslips were sealed with nail polish. 

Gravid females were examined in whole mounts of individual segments (*n* = 8 females) or in paraffin section (*n* = 5). Whole-mounted segments were processed as for embryos. For paraffin sectioning, females were placed in filtered seawater for 24 h to allow the gut to void of sand and then fixed and dehydrated as described for egg capsules. After females were embedded in paraffin, they were sectioned at 10 *μ*m and 5-6 sections from each female placed on poly-L-lysine coated slides. Sections were deparaffinized in xylene, rehydrated to PT, and processed as described for egg capsules. Sections were ringed with a liquid blocker pen to avoid loss of reagents, and the immunohistochemistry protocol was done in a humid, sealed chamber.

Negative controls were processed using the typical protocol but with the primary antibody replaced with 2% BSA/PT during the first incubation, followed by labeling as usual with FITC- or TRITC-conjugated secondaries during the second incubation. Epifluorescence was not detected in the negative controls.

Samples were examined using a Zeiss Axioplan II compound fluorescence microscope and micrographs taken using an SPOT-2 camera (Diagnostic Instruments, Inc.). Micrographs were adjusted for size and contrast using Corel PhotoPaint 11.0. 

## 3. Results and Discussion

### 3.1. Detection of Histones (Antihistone 1 and Core Histones)

**Figure 1:**
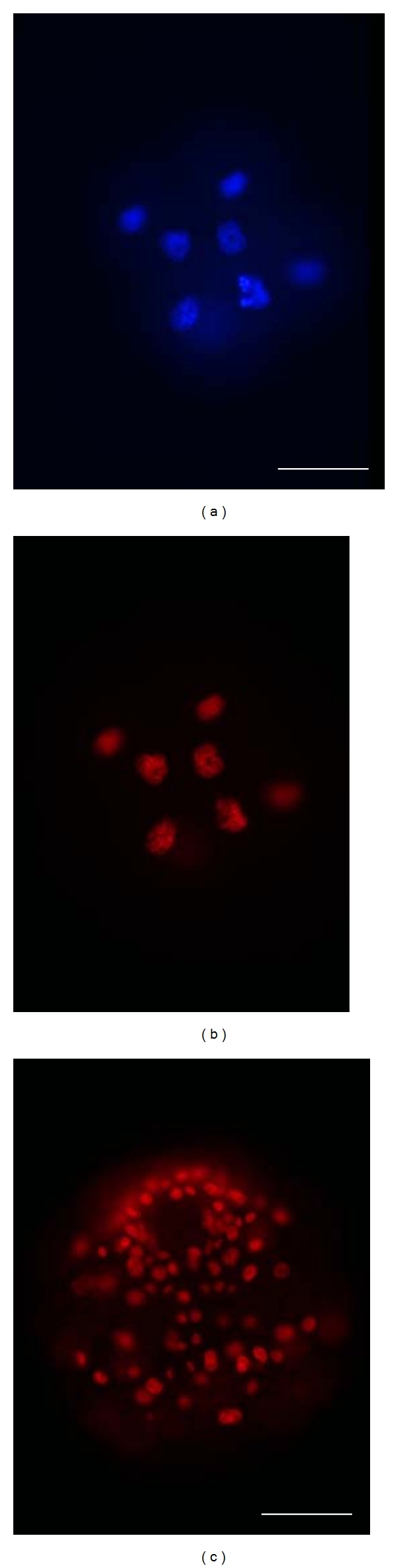
Distribution of core histones in embryos of *Polydora cornuta*. (a, b) Companion micrographs of an eight-cell embryo from an A-brood, showing the distribution of DNA (a, DAPI) and histones (b, TRITC-conjugated anti-histone). (c) Gastrula from a P-brood labeled with TRITC-conjugated antihistone. Scale bars = 50 *μ*m.

Epifluorescence of TRITC-conjugated antihistone indicated that histones were present in the nuclei of all cells throughout development (Table 1). TRITC epifluorescence colocalized with that of DAPI, providing evidence that the TRITC signal was restricted to nuclear chromatin (Figures 1(a) and 1(b)). TRITC-conjugated antihistone was detected in all cells of embryos and larvae throughout development and is shown here for a gastrula from a P-brood (Figure 1(c)). These results demonstrate that this antibody appears to recognize and bind to worm antigens, and also that we can detect histones in all blastomeres, even at early stages when the blastomeres are very yolky. 

### 3.2. Antihistone H3 Acetyl Lys14

**Figure 2:**
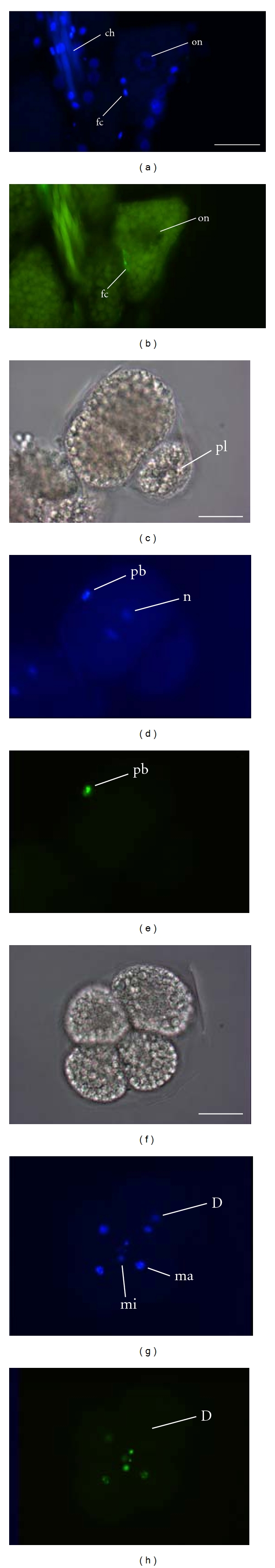
H3K14 acetylation in early development of *Polydora cornuta*. (a, b) Companion micrographs of an oocyte inside the coelom of a female in paraffin section showing nuclear DNA (a, DAPI) and nuclei that are acetylated at H3K14 (b, FITC-conjugated anti-H3K14ac). The remaining images are bright field (left) and companion images showing DNA (DAPI, middle) and nuclei with H3K14ac (FITC-conjugated anti-H3K14ac, right). (c–e) Two-cell stage with polar bodies and a polar lobe (P-brood). (f–h) Eight-cell embryo, shown from the animal pole (P-brood). ch: chaetae, D: D macromere, fc: follicular cell, ma: macromere, mi: micromere, n: nucleus, on: oocyte nucleus, pb: polar body, and pl: polar lobe. Scale bars = 50 *μ*m.

Acetylation of H3K14 was not detected in oocytes located within the coelom of gravid females although it was detected in a few of the follicular cells associated with the oocytes (Figures 2(a) and 2(b)). The earliest embryo that was surveyed for H3K14ac was at a two-cell cleavage stage. H3K14ac was not detected in blastomeres although it was evident in the polar bodies (Figures (2(c)–2(e); Table 1). Four-cell embryos were similar: H3K14ac was not detected in blastomeres, but it was detected in polar bodies (not shown). H3K14ac was first detected in blastomeres in embryos that were entering the eight-cell stage. In these embryos, H3K14ac was present in all blastomeres except the large D macromere (Figures 2(f)–2(h), shown in an embryo in which the D blastomere is undergoing mitosis; H3K14ac was detected in mitotically active cells in other embryos, described below). Acetylation of H3K14 was also absent in the D blastomere of 12- to 16-cell embryos (not shown). By late cleavage (roughly 32 cells), H3K14ac appeared to be present in most, if not all, blastomeres although epifluorescence in the inner, yolky macromeres was sometimes difficult to observe.

**Figure 3:**
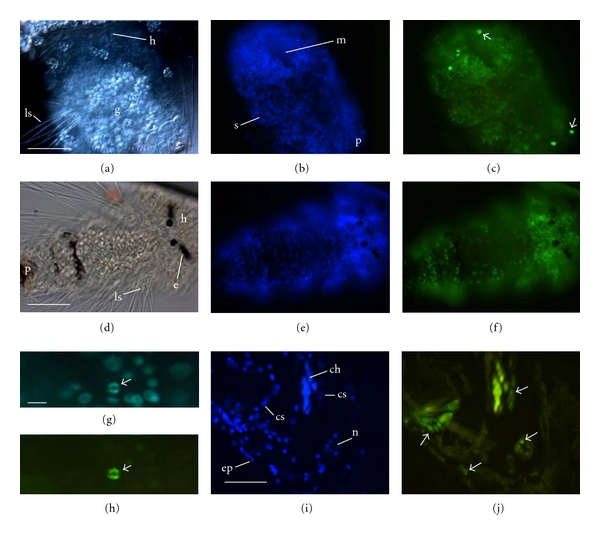
H3K14 acetylation in larvae and adults of *Polydora cornuta*. (a–c) Companion micrographs of the ventral surface of a three-chaetiger larva (A-brood) in bright field (a), showing nuclear DNA (b, DAPI), and showing nuclei that are acetylated at H3K14 (c, FITC-conjugated anti-H3K14ac). Note the strong epifluorescence that is typical of cells of the mouth and pygidium (small arrows in c). (d–f) Dorsal view of a four-chaetiger larva (A-brood) in bright field (d), with DAPI (e) and with FITC-conjugated anti-H3K14ac (f). (g, h) Images of an ectodermal cell from a gastrula (P-brood) that is undergoing mitosis, double labeled with DAPI (g) and FITC-conjugated anti-H3K14ac (h). (i, j) Paraffin section through the body wall of a female that is double labeled with DAPI (i) and FITC-conjugated anti-H3K14ac (j). cs: chaetal sac, ch: chaetae, e: eye, ep: epidermis, g: gut, h: head, ls: larval spines, m: mouth, n: nephridium, s: segment, and p: pygidium. Arrows indicate the presence of FITC-conjugated H3K14ac in the indicated cells. Scale bars = 10 *μ*m (g, h) or 50 *μ*m (a–f, i, j).

In gastrulae, H3K14ac was detected as weak epifluorescence throughout the epidermis and as strong epifluorescence in a few cells associated with the mouth (shown below). In trochophores and early larvae, bright epifluorescence was detected in the mouth and also on the ventral surface and pygidium (Figures 3(a)–3(c), shown for a three-chaetiger adelphophagic larva). As larvae developed, this pattern of H3K14ac was retained: weak epifluorescence was detected throughout the epidermis (Figures 3(d)–3(f), dorsal view shown in a four-chaetiger adelphophagic larvae), and strong epifluorescence was present in a few cell lineages, specifically the mouth, ventral cells, and pygidium. Bright epifluorescence was also observed in cells during mitosis, indicating that H3K14ac persists or is restored through karyokinesis (Figures 3(g) and 3(h)). Older larvae had a similar distribution of H3K14ac-positive cells (not shown). In contrast, gravid females had detectable levels of H3K14ac in relatively few cells, including scattered cells of the epidermis, nephridia, and chaetal sacs (Figures 3(i) and 3(j)). Importantly, the differential brightness observed in specific cell lineages was consistent among young from the same egg capsules and across multiple (in some cases, up to four) broods per ontogenetic stage.

Histone acetylation is generally associated with transcription and in eukaryotes is common in undifferentiated cells, while differentiated cells often contain hypoacetylated chromatin [26]. Our observations fit with that general pattern as H3K14ac was acquired in most blastomeres (i.e., all except the D macromere) in early development (around the 8-cell stage), rapidly growing larvae had detectable levels of H3K14ac throughout the epidermis, and the relative number of H3K14ac-positive cells decreased in adults. One result that differed from that reported in model systems (specifically, mammals and *Drosophila*) is the lack of detectable H3K14ac in oocytes and early embryos (2- to 4-cell stages). Histone acetylation is important in oogenesis in mammals [1] and while lack of H3K14ac is reported in mammalian zygotes, H3K14ac is often restored in cleavage [27]. H3K14ac is also important in meiosis in oocytes of *Drosophila* and has been shown to vary at different points in the meiotic cycle [28]. Our detection of H3K14ac in polar bodies is consistent with Endo et al. (2005) who detected a strong signal of histone acetylation in polar bodies in mammals [1]. In *P. cornuta*, it appears that H3K14ac is important in at least some aspects of meiosis, as it was detected in polar bodies and suggests a potential pathway by which polar bodies may be determined.

The onset of acetylation of H3K14 occurred in early embryos (8-cell stage), when it was detected in all blastomeres except for the D macromere; this pattern was retained at least through the sixteen-cell stage but was difficult to follow in later development (from 32 cells on) given the techniques used here. In polychaetes, the D macromere gives rise to most of the segmented tissue including ectoderm and mesodermal derivatives [29]. Lack or delayed onset of acetylation of H3K14 in the D blastomere suggests a delay in transcription of some genes within this lineage, but this remains to be confirmed.

### 3.3. Antihistone H3 Monomethyl Lys9

**Figure 4:**
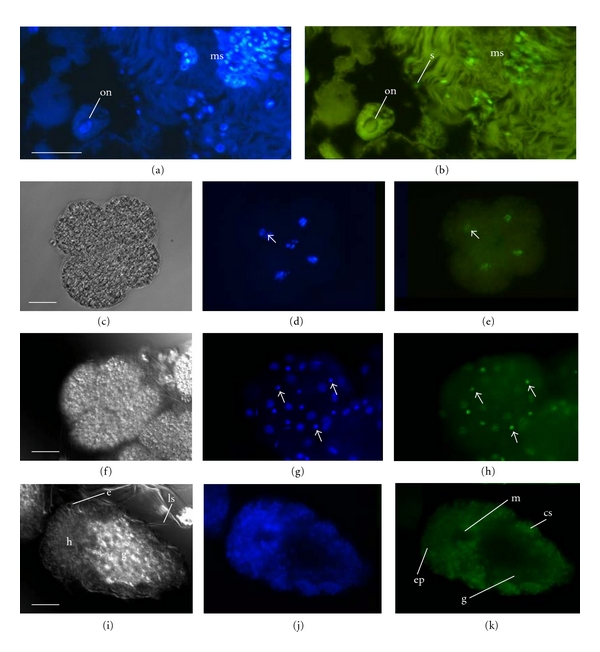
H3K9 monomethylation in *Polydora cornuta*. (a, b) Companion micrographs of an immature oocyte inside the coelom of a female in paraffin section showing nuclear DNA (a, DAPI) and nuclei that are monomethylated at H3K9 (b, FITC-conjugated anti-H3K9me). The remaining images are bright field (left) and companion images showing DNA (DAPI, middle) and nuclei with H3K9me (FITC-conjugated anti-H3K9me, right) for embryos and larvae from planktotrophic broods. (c–e) Eight-cell embryo shown from the animal pole. The small arrow indicates the presence of H3K9me in a dividing blastomere. The micromeres are out of the plane of focus and are difficult to see in (e). (f–h) Blastula, shown from the animal pole. The small arrows indicate the ring of hypermethylated micromeres surrounding the presumptive head. (i–k) Ventral view of a three-chaetiger larva. H3K9me is visible throughout the epidermis, chaetal sacs, and gut. cs: chaetal sac, e: eye, ep: epidermis, g: gut, h: head, ls: larval spines, m: mouth, ms: muscle, on: oocyte nucleus, and s: septa. Scale bars = 50 *μ*m.

H3K9me was not detected in oocytes located within the coelom of gravid females (Figures 4(a) and 4(b)). The earliest stage of development in which H3K9me was detected was the eight-cell stage where it was detected in all blastomeres (Figures 4(c)–4(e); note that the distribution of H3K9me is shown for planktotrophic embryos and larvae throughout this section; Table 1). In blastulae, H3K9me was detected as weak epifluorescence in all blastomeres but gave a characteristically bright signal in a ring of cells around the presumptive head (Figures 4(f)–4(h)), a distribution pattern that was retained in gastrulae. We interpreted this bright signal as due to increased histone modification (i.e., hypermethylation) rather than altered nuclear structure (i.e., micromeres giving brighter signal in their nuclei simply because they were small and concentrated). Our interpretation is founded on the argument that other micromeres (e.g., the two in the centre of the blastula in Figures 4(g) and 4(h)) also had small and concentrated nuclei, but did not possess the same bright signal as those forming the ring around the presumptive head. 

In trochophores and metatrochophores, moderate levels of H3K9me were detected throughout the ectoderm with bright epifluorescence in the head and laterally in the region of the presumptive chaetal sacs. Epifluorescence was also detected in the mesoderm and endoderm (demonstrated below). Early larvae had moderate FITC signal throughout the epidermis, chaetal sacs and mesoderm, and weak epifluorescence in the gut (shown in a three-chaetiger larva; Figures 4(i)–4(k)), a pattern of distribution that was retained at least to the six-chaetiger stage. In gravid females, H3K9me was detected in ectodermal and mesodermal derivatives including many epidermal cells, nephridia, muscle, and a few cells of the chaetal sacs (shown for muscle and septa; Figures 4(a) and 4(b)). 

These observations suggest that the distribution of H3K9me is in many ways similar to H3K14ac; H3K9me was not detected in oocytes, it was first detected in early cleavage, and it was widely distributed in cells of embryos and larvae and varied in intensity among cell lineages (e.g., in the blastula stage), and H3K9me-positive cells decreased in distribution in adults. Here, we interpret the presence or relative intensity of H3K9me as indicating a change in histone modifications (onset, loss, or hypermethylation) rather than specifically indicating gene activation or repression, as H3K9me has been associated with both [19, 25, 30, 31]. Differences among cell lineages in epifluorescence intensity were consistent from blastulae to larvae, with low levels of epifluorescence throughout the ectoderm and bright signal in some nuclei of the head. This suggests that hypermethylation occurs in these cells and may affect differential levels of gene expression as differentiation occurs. Adults showed a decrease in methylation of H3K9; in larvae, H3K9me was broadly found in most, if not all, cells of the epidermis, mesoderm, and, gut but in adults, H3K9me was restricted to relatively few cells of the epidermis and mesodermal derivatives. This suggests that a transition in histone modifications occurs between larvae and adults that involves a shift from detectable levels of H3K9me in most cells to methylation in few cell lineages only and is consistent with loss of methylation of H3K9 during differentiation that has been observed elsewhere [19]. 

### 3.4. Antihistone H3 Dimethyl Lys4

**Figure 5:**
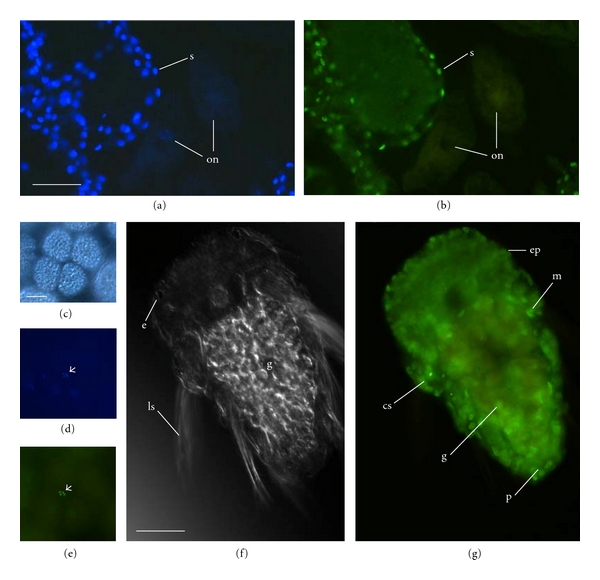
H3K4 dimethylation in *Polydora cornuta*. (a, b) Companion micrographs of an oocyte inside the coelom of a female in paraffin section showing nuclear DNA (a, DAPI) and nuclei that are dimethylated at H3K4 (b, FITC-conjugated anti-H3K4me2). Note that H3K4me2 was not detected in the oocyte nucleus. (c–e) Early cleavage stages of a planktotrophic embryo in bright field (c) and with epifluorescence for DAPI (d) and FITC-conjugated H3K4me2 (e). The small arrows indicate the presence of H3K4me2 in the micromeres. (f, g) Three-chaetiger larva from a P-brood in bright field (f) and with epifluorescence for FITC-conjugated H3K4me2 (g). e: eye, ep: epidermis, g: gut, ls: larval spines, m: mesoderm, on: oocyte nucleus, s: septa, and p: pygidium. Scale bars = 50 *μ*m.

H3K4me2 was not detected in oocytes located within the coelom of gravid females (Figures 5(a) and 5(b)). H3K4me2 was detected in both micromeres and macromeres in early cleavage although epifluorescence was difficult to detect in the macromeres because of the large amount of yolk in these cells (six- to eight-cell embryos; Figures 5(c)–5(e); Table 1). In blastulae and gastrulae, H3K4me2 was present in most, if not all, blastomeres. At the trochophore stage, H3K4me2 was detected throughout the ectoderm and also in some of the deeper cells of the underlying mesoderm (demonstrated below). The distribution of H3K4me2 was similar in early larvae (i.e., three to four chaetigers in length) and was generally detected throughout the epidermis and underlying muscle and was also detected as weak epifluorescence in the developing gut (Figures 5(f) and 5(g)). Only adelphophagic larvae were observed at later ontogenetic stages. In later larval development (i.e., five chaetigers) and at hatching (roughly twelve chaetigers), adelphophagic larvae still had strong FITC signal associated with the epidermis and in the underlying muscle (not shown). In gravid females, H3K4me2 was detected throughout the epidermis, septa, and muscle but not the gut (shown for septa; Figure 5(a) and 5(b)).

These observations suggest that H3K4me2 was similar in distribution to H3K14ac and H3K9me: H3K4me2 was not detected in oocytes, had an onset in early cleavage, was broadly distributed in embryos and larvae, and was detected in adults in specific tissues only. The major difference between H3K4me2 and the modifications described above was the lack of hypermethylation in specific cell lineages. These observations suggest that widespread changes in H3K4me2 occur at roughly the eight-cell stage (i.e., onset) and possibly also with metamorphosis (i.e., change in tissue-specific expression). As with H3K9me, the specific functional implications of H3K4me2 are not yet known for *P. cornuta*, as dimethylation of H3K4 is associated with varying transcriptional activity depending on interactions with other histone modifications as differentiation occurs [20, 32].

### 3.5. Antihistone H4 Dimethyl Lys20

We attempted to detect H4K20me2 in female tissue (specifically the body wall and palps), in trochophores, and in three-chaetiger larvae. H4K20me2 was not detected at any of these ontogenetic stages, and therefore, our search for it was discontinued.

### 3.6. Changes in Histone Modifications throughout Development

In many metazoans, histone modifications are re-programmed during meiosis and embryos gradually acquire modifications during differentiation [33]. Our results suggest that this general pattern also occurs in polychaetes. H3K14ac, H3K9me, and H3K4me2 were not detected in oocytes, but all three were detected in early cleavage embryos (at roughly the eight-cell stage in the planktotrophic morph), had a widespread distribution in larvae within the derivatives of certain germ layers, and were detected in adults but in specific tissues only. 

Collectively, these observations suggest that global changes in gene expression occur at about the eight-cell stage with the onset of three modifications that affect gene transcription (i.e., H3K14ac, H3K9me, and H3K4me2). The onset of histone modifications was consistent but not uniform among blastomeres; for example, the onset of H3K14ac was delayed in the D blastomere relative to other cells of the same embryo. While the importance of histone modifications in the early development of marine invertebrates has not received much attention, the importance of histone variants has been demonstrated by Arenas-Mena and colleagues for the polychaete *Hydroides elegans* and the sea urchin *Strongylocentrotus purpuratus* [34]. Both species express the histone variant H2A.Z in early cleavage where it is specifically associated with undifferentiated cells, and as cellular differentiation occurs in larvae, the expression of H2A.Z declines [34]. Our observations suggest that histone modifications (in addition to histone variants) may be associated with determination of cell fate in polychaetes, given the common onset of three modifications in early cleavage in *P. cornuta*. Additionally, histone modifications are associated with differentiation as they were also detected in specific larval and adult tissues. 

The potential for histone modifications to be associated with tissue differentiation is supported by the presence of hyperacetylation of H3K14 in cells of the mouth and pygidium of larvae, of hypermethylation of H3K9 in cells of the presumptive head of embryos (blastulae and gastrulae), and of the restriction of some modifications to specific organs (e.g., H3K9me and H3K4me2 were detected in the larval gut, but H3K14ac was not). Thus, lineage-specific modifications also occur and suggest that histone modifications may influence not only early specification of cell fate but also cell differentiation as tissues (such as the gut) specialize and become functional in larvae.

All three histone modifications had patterns of distribution that differed between larvae and adults. In larvae, H3K14ac was broadly distributed throughout the epidermis, and H3K9me and H3K4me2 were detected throughout derivatives of ectoderm, mesoderm, and endoderm. In contrast, the distribution of all three modifications was restricted in adults, in terms of being detected in relatively few cells within a tissue (e.g., H3K14ac in the adult epidermis) or no longer being present at detectable levels (e.g., H3K4me2 in the adult gut). This suggests that a transition in the histone code may occur as larvae undergo metamorphosis. Metamorphosis was not a focus of this study, but this general pattern suggests two hypotheses. One is that histone modifications affect a change in gene expression that correlates with changes in growth from rapidly growing larvae to more slowly growing adults. The other hypothesis is that changes in histone modifications correlate with a developmental reprogramming at metamorphosis. Both hypotheses have merit. Most larval tissues contribute directly to adult tissues in spionid polychaetes, suggesting that the first hypothesis may be more valid, but settlement involves widespread behavioural and morphological changes; thus, the potential for a global reprogramming of gene expression at metamorphosis is also to be considered. 

## 4. Conclusions

We surveyed histone modifications in the development of a polychaete, *Polydora cornuta*, using immunohistochemistry. We found that three of the four tested primary antibodies for histone modifications appeared to recognize and bind to antigens of this species. H3K14ac, H3K9me, and H3K4me2 colocalized with DAPI and were consistently detected throughout development. The fourth primary antibody, H4K20me2, did not react with the tissue. The three detected modifications collectively suggest that these histone modifications are first present in early cleavage, are widely distributed throughout larval development, and also are found in some adult tissues but with a more restricted distribution. The observed common onset in histone modifications suggests that a global change or activation of gene expression occurs in early embryos. Two modifications showed a generally low level of epifluorescence in most cells but a very strong signal in a few cell lineages, indicating a role in tissue differentiation. Finally, differences in the distribution of three modifications between larvae and adults suggest a second transition in histone modifications may occur at metamorphosis. Although preliminary, this research indicates that histone modifications are present in a marine invertebrate and show characteristic changes with tissue differentiation and also with specific life stages.

## Figures and Tables

**Table 1:** Summary of histones and epigenetic modifications during development of *Polydora cornuta*. The modifications listed were common to both morphs (i.e., were observed in both adelphophagic and planktotrophic young). + = modification present; − = modification absent; blank = no sample. h = head, m = mouth, p = pygidium, vc = ventral cells.

											
Modification	Developmental stage										
	Oocyte	2–4 cells	8 cells	32 cells	Blastula	Gastrula	Trochophore	Metatrochophore	3 chaetigers	5–12 chaetigers	Adult
Histones	+	+	+	+	+	+	+	+	+	+	+
H3K14ac	−	−	+ (not D)	+	+	+* (m)	+* (m, vc, p)	+* (m, vc, p)	+* (m, vc, p)	+* (m, vc, p)	Some tissues
H3K9me	−	−	+	+	+* (h)	+* (h)	+	+	+	+	Some tissues
H3K4me2	−		+	+	+	+	+	+	+	+	Some tissues
H4K20me2							−		−		−

*interpreted as hyperacetylation or hypermethylation in indicated cell lineages (see text).
